# Redox Signaling as a Therapeutic Target to Inhibit Myofibroblast Activation in Degenerative Fibrotic Disease

**DOI:** 10.1155/2014/131737

**Published:** 2014-02-20

**Authors:** Natalie Sampson, Peter Berger, Christoph Zenzmaier

**Affiliations:** ^1^Division of Experimental Urology, Department of Urology, Innsbruck Medical University, Anichstrasse 35, A-6020 Innsbruck, Austria; ^2^Institute for Biomedical Aging Research, University of Innsbruck, 6020 Innsbruck, Austria; ^3^Department of Internal Medicine III, Innsbruck Medical University, Anichstrasse 35, A-6020 Innsbruck, Austria

## Abstract

Degenerative fibrotic diseases encompass numerous systemic and organ-specific disorders. Despite their associated significant morbidity and mortality, there is currently no effective antifibrotic treatment. Fibrosis is characterized by the development and persistence of myofibroblasts, whose unregulated deposition of extracellular matrix components disrupts signaling cascades and normal tissue architecture leading to organ failure and death. The profibrotic cytokine transforming growth factor beta (TGF**β**) is considered the foremost inducer of fibrosis, driving myofibroblast differentiation in diverse tissues. This review summarizes recent *in vitro* and *in vivo* data demonstrating that TGF**β**-induced myofibroblast differentiation is driven by a prooxidant shift in redox homeostasis. Elevated NADPH oxidase 4 (NOX4)-derived hydrogen peroxide (H_2_O_2_) supported by concomitant decreases in nitric oxide (NO) signaling and reactive oxygen species scavengers are central factors in the molecular pathogenesis of fibrosis in numerous tissues and organs. Moreover, complex interplay between NOX4-derived H_2_O_2_ and NO signaling regulates myofibroblast differentiation. Restoring redox homeostasis via antioxidants or NOX4 inactivation as well as by enhancing NO signaling via activation of soluble guanylyl cyclases or inhibition of phosphodiesterases can inhibit and reverse myofibroblast differentiation. Thus, dysregulated redox signaling represents a potential therapeutic target for the treatment of wide variety of different degenerative fibrotic disorders.

## 1. Introduction: Fibrosis and Degenerative Fibrotic Diseases

The wound healing response in which damaged/dead cells are replaced following acute injury (such as infection, autoimmune reaction, or mechanical injury) is essential to maintain tissue architecture and function [1–4]. However, if the healing process continues unchecked, for example, due to repeated/chronic injury, fibrosis ensues as characterized by substantial deposition and remodeling of the extracellular matrix (ECM) and permanent scar tissue formation, which destroys correct tissue architecture and may ultimately lead to organ failure and death [1–4].

There are numerous degenerative fibrotic diseases, including multisystemic disorders such as systemic sclerosis, chronic graft versus host disease, and nephrogenic systemic fibrosis as well as organ-specific diseases, for example, cardiac fibrosis, idiopathic pulmonary fibrosis (IPF), intestinal fibrosis, liver cirrhosis, progressive kidney disease, macular degeneration, and benign prostatic hyperplasia (BPH) [1–3, 5–12]. In addition, a multitude of disorders with prominent tissue remodeling also have a significant fibrotic component, including asthma, atherosclerosis, and the reactive stromal response to solid tumors, such as breast, liver, and prostate cancer [13–16]. Thus, it is perhaps not surprising that approximately 45% of the mortality in Western nations is attributed to fibrotic diseases, a figure that is certainly even higher in less developed countries [12].

Despite the considerable morbidity and mortality caused by fibrosis, there are currently no effective treatments for many of these diseases and no approved antifibrotic therapies. In part, this is due to our current lack of knowledge regarding (i) the precise etiology of the initiating injury/infection and (ii) the mechanisms that drive fibrosis progression. Thus, a better understanding of the molecular pathways underlying fibrosis and the initiating signals/causes is urgently required for the development of effective therapeutic strategies. This review focuses on accumulating evidence that redox signaling plays a fundamental and integral role in the molecular pathogenesis of fibrosis in many different tissues and organs and as such represents a potential therapeutic target for the treatment of wide variety of different fibrotic disorders.

## 2. The Myofibroblast: Biology, Origin, and Role in Fibrosis

Fibrotic diseases are clearly distinct in their etiology and clinical manifestation. Nonetheless, fibrogenesis in most organs and tissues progresses in a remarkably similar manner, characterized in particular by the development and persistence of large numbers of myofibroblasts [3, 7, 9, 12]. During the normal wound healing response, myofibroblasts accumulate to promote wound closure by virtue of their contractile and ECM- and growth factor-secreting properties, with the latter serving to attract epithelial cells, a process termed “reepithelialization”. Normal tissue function and architecture are restored upon completion of reepithelialization via poorly understood mechanisms that result in massive apoptosis of myofibroblasts and vascular cells, which are subsequently cleared from the wound site [7, 17, 18]. Tissue and organ fibrosis are thought to arise from failure of myofibroblast apoptosis during wound healing [3, 19]. Again, however, the mechanisms underlying this apparent “apoptosis-resistant” myofibroblast phenotype remain ill-defined [20]. The resulting persistent myofibroblast activation leads to excessive ECM deposition, altered growth factor signaling and consequently cellular proliferation, progressive remodeling and destruction of normal tissue architecture, organ dysfunction, and failure [3, 19, 21]. Thus, the myofibroblast is widely considered the main effector cell of fibrosis and thereby a major therapeutic target.

Myofibroblasts are a specialized cell type that combines the ECM-producing characteristics of fibroblasts with the cytoskeletal and contractile properties of smooth muscle cells (SMCs) as reviewed recently [2]. Myofibroblasts are defined by (i) their *de novo* expression of alpha-smooth muscle cell actin (*α*-SMA, encoded by the gene *ACTA2*) in stress fibers and (ii) contractile force. The cellular origin of myofibroblasts remains somewhat controversial but may differ depending on the organ and/or the initiating stimulus (reviewed [2, 22]). Myofibroblasts have been described to originate from differentiation of vascular SMCs, bone marrow-derived fibrocytes, hepatic stellate cells, resident epithelial cells via epithelial-to-mesenchymal transition, and endothelial cells via endothelial-to-mesenchymal transition [3, 23]. However, although these cell types undergo differentiation into myofibroblasts *in vitro*, the extent of their contribution to the myofibroblast pool *in vivo *is the subject of considerable debate. Rather, it is widely accepted that myofibroblasts predominantly originate from the differentiation of local tissue fibroblasts [23].

**Figure 1:**
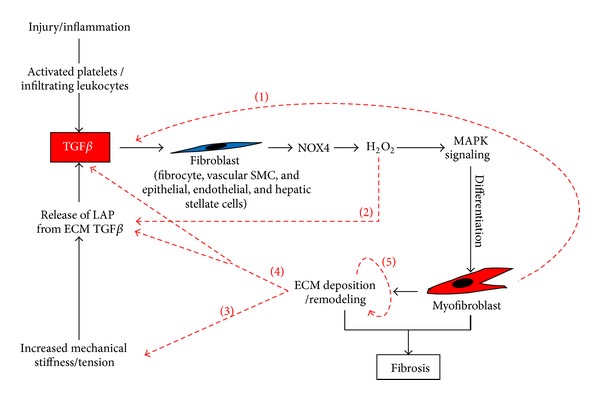
Feed-forward loop of TGF*β* activation and myofibroblast differentiation in fibrosis. Upon injury activated platelets, infiltrating inflammatory and vascular cells secrete TGF*β*, which acts on local fibroblasts and other precursor cells (e.g., hepatic stellate cells, fibrocytes) inducing their production of NOX4-derived H_2_O_2_. Consequently, downstream MAPK signaling cascades are activated resulting in differentiation into myofibroblasts, whose production of ECM components facilitates wound closure. Prolonged injury or inflammation leads to persistent myofibroblast activation via a feed-forward loop driven by several different factors. For example, myofibroblasts themselves secrete and produce large amounts of active TGF*β* and thereby generate an autocrine feed-forward loop that is characteristic of persisting myofibroblast activity (1) [27]. Activation of latent TGF*β* in ECM deposits via dissociation of latency associated peptide (LAP) is promoted by various mechanisms, including direct oxidative modification (2) [29–31]. Thus, NOX4-derived H_2_O_2_ may drive myofibroblast differentiation not only by oxidative modulation of MAPK signaling cascades that culminate in downstream transcriptional programs of differentiation [26], but also via its ability to freely diffuse across biological membranes and oxidatively modulate components in the extracellular space. Myofibroblasts also secrete high levels of ECM components. The resulting increase in mechanical tension and tissue stiffness can activate ECM-bound latent TGF*β* due to mechanical pulling of LAP by specific integrins at the myofibroblast cell surface (3) [32]. Thereby, TGF*β* is released and activated from the latent complex, which in turn drives further myofibroblast contraction and differentiation as well as ECM deposition [25]. In addition to this physical mechanism of TGF*β* activation by the remodeled ECM, components of the remodeled ECM can modulate TGF*β* signaling in a biochemical manner (4), for example, latent TGF*β* binding proteins, fibrillins, fibulins, fibronectin, and proteoglycans (reviewed [33]). Moreover, a number of targets downstream of TGF*β* signaling provide feedback modulation of the ECM either directly or indirectly, for example, thrombospondin-1 (TSP-1), collagens/ECM components themselves, and ECM remodeling components such as matrix metalloproteinases (5) (MMP2, -9), plasminogen activator inhibitor (PAI-1), and tissue inhibitors of metalloproteinases (TIMPs) [26]. Thus, the stiffened/remodeled ECM together with autocrine production of TGF*β* and NOX4-derived H_2_O_2_ actively perpetuate TGF*β* signaling and myofibroblast differentiation leading to fibrosis.

Fibroblast-to-myofibroblast differentiation occurs via a two-step process. Following injury or during chronic inflammation, changes in mechanical tension of the ECM are transmitted to the fibroblast cytoskeleton via RhoA/ROCK signaling [24]. Consequently, fibroblasts adopt an “activated” phenotype (termed “protomyofibroblast”) and deposit new ECM components [25]. Soluble factors and cytokines, in particular the splice variant ED-A of cellular fibronectin and profibrotic cytokine TGF*β*, which are produced initially by platelets and infiltrating leukocytes at the wound site, are major inducers of fibroblast-to-myofibroblast differentiation [25]. However, protomyofibroblasts and myofibroblasts themselves also secrete and activate TGF*β* thus generating an autocrine feed-forward loop driving continued myofibroblast differentiation [26, 27] (Figure 1). It may be noted, however, that, although inflammation frequently occurs prior to fibrosis, fibrogenesis can also occur independently of inflammatory mechanisms indicating that inflammation is not always the driving initiator [28].

Although several TGF*β*-independent mechanisms of fibrosis have been described, such as interleukins 4 and 13 and platelet-derived growth factor (reviewed [34, 35]), TGF*β*1 is widely considered the foremost inducer of fibrosis and drives myofibroblast differentiation in cells of diverse histological origin, including breast, skin, prostate, kidney, heart, lung, and liver [36–42]. Consistently, elevated TGF*β*1 levels and signaling are observed in many fibrotic disorders [19, 43–50]. TGF*β*1 exerts its effects via downstream activation of canonical Smad2/3 signaling or via noncanonical Smad-independent activation of mitogen-activated protein kinase (MAPK) and PI3 kinase/Akt pathways [2, 26, 51]. Collectively, signaling via these pathways leads to ECM deposition and secretion of paracrine- and autocrine-acting growth factors [26, 52]. Notably, the ECM can directly bind to and release growth factors; for example, heparan sulfate can bind to and release fibroblast growth factor 2 [53]. On the one hand, such interactions sequester growth factors thereby protecting them from degradation but can also enhance their bioactivity due to increased half-life [54]. Moreover, indirect interactions are required for signal transduction of some growth factors; for example, integrin binding is necessary for induction of angiogenesis by vascular endothelial cell growth factor [55]. Thus, remodeling and enhanced deposition of ECM in fibrosis contributes to disease pathogenesis not only by disrupting normal tissue architecture but also by modulating cellular signaling cascades (Figure 1).

TGF*β* undoubtedly plays a pivotal role in pathogenic fibrogenesis. Therapeutic approaches designed to interfere with downstream TGF*β* signaling processes that culminate in myofibroblast activation may represent an alternative viable strategy for the treatment of fibrotic disease. In this respect, a convincing body of data implicates dysregulated redox signaling by NADPH oxidase 4 (NOX4) and nitric oxide (NO) in the pathophysiology of fibrosis.

## 3. Signaling by NOX4-Derived Reactive Oxygen Species in the Regulation of Myofibroblast Differentiation

High levels of free radicals can result in nonspecific oxidative damage to cell structures and biomolecules. However, when produced in a regulated manner, reactive oxygen species (ROS), NO, and reactive nitrogen species play a critical role as biological second messengers in a variety of cellular processes, including myofibroblast differentiation [56]. NADPH oxidase (NOX) enzymes are unique in that ROS production is their primary and sole function [57]. This is in contrast to ROS-producing enzyme systems such as xanthine oxidase or uncoupled endothelial NO synthase, whose production of ROS occurs secondary to their primary function. The seven members of the NOX family catalyze the transfer of electrons across biological membranes from NADPH to oxygen thereby generating superoxide (O_2_^•−^) [58]. However, the major detected product and primary effector ROS of the constitutively active NOX4 is hydrogen peroxide (H_2_O_2_), although this most likely is a result of rapid superoxide dismutation [59–61]. It is thought that a highly conserved histidine residue within the E-loop of NOX4 promotes rapid dismutation of superoxide before it leaves the enzyme [61], although this aspect of NOX4 biology requires further clarification. Irrespectively, the greater stability but lower reactivity of H_2_O_2_ compared to superoxide is consistent with a signaling function of NOX4-derived ROS [26, 62, 63]. NOX-derived ROS exert their signaling functions by modulating biological activity of target proteins such as transcription factors, MAPKs, protein tyrosine phosphatases (PTPs), and protein tyrosine kinases via reversible oxidation of thiol groups of low pKa cysteine residues [63, 64].

**Figure 2:**
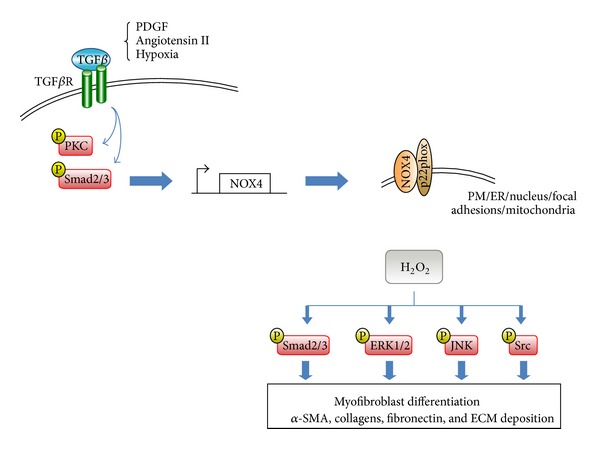
Signaling pathways that activate NOX4 and downstream targets of NOX4-derived ROS in myofibroblasts. NOX4 activity is predominantly regulated at the transcriptional level [59, 65]. TGF*β* is one of the main inducers of *NOX4* during myofibroblast activation. Additionally, hypoxia, angiotensin II, and platelet-derived growth factor (PDGF) have also been shown to activate *NOX4* expression leading to myofibroblast activation; however, this most likely occurs as a result of their indirect activation of TGF*β* signaling [75–77]. Upon binding of TGF*β* ligand, heteromeric complexes of TGF*β* receptor type I and type II recruit and activate the canonical signal transducers Smad2/3 as well as less well understood noncanonical signal transducers, such as mitogen-activated protein kinases (MAPKs) and protein kinase C (PKC). TGF*β* signal transducers subsequently activate the transcription of target genes that include *NOX4*. TGF*β*-mediated induction of NOX4 expression has largely shown to be Smad2/3-dependent [39, 40]; however, PKC has also been implicated in TGF*β*-dependent upregulation of NOX4 [78]. The subcellular localization of NOX4 appears to be cell-, tissue-, and perhaps even context-specific with its reported localization to the plasma membrane (PM), endoplasmic reticulum (ER), nucleus, focal adhesions, and mitochondria [62]. NOX4 requires the cofactor p22^phox^ for production of ROS, of which predominantly H_2_O_2_ is detected [59–61]. NOX4-derived H_2_O_2_ activates signaling intermediates such as Smad2/3, ERK1/2, JNK and Src [26, 39, 40, 77, 79–81], which subsequently induce the transcription of downstream target genes, such as *α*-smooth muscle cell actin (*α*-SMA), collagens, and fibronectin leading to ECM deposition and myofibroblast differentiation/activation.

**Figure 3:**
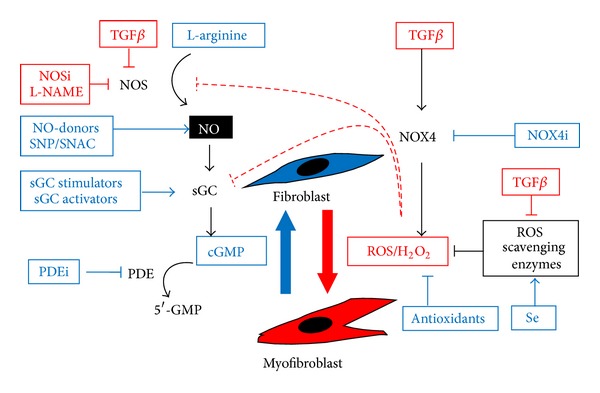
Potential therapeutic targeting of myofibroblast differentiation. Myofibroblast differentiation plays a central role in the etiology of fibrosis. TGF*β*, which is widely considered the foremost inducer of fibrosis, induces NOX4 resulting in a persistent prooxidant shift in intracellular redox homeostasis mediated via ROS (in particular H_2_O_2_), which modulates downstream phosphorylation signaling cascades and transcriptional events culminating in fibroblast-to-myofibroblast differentiation. The concomitant downregulation of selenium (Se)-dependent ROS scavenging enzymes by TGF*β* further potentiates NOX4-derived ROS signaling. In parallel, TGF*β* and H_2_O_2_ attenuate NO signaling, which is associated with the fibroblast phenotype, via attenuation of NOS and sGC activities. Likewise, NOS inhibitors (NOSi/L-NAME) attenuate NO signaling and aggravate fibrosis. Fibroblast-to-myofibroblast differentiation and subsequent tissue fibrosis are reversible processes. Thus, targeting persistent NOX4-derived ROS levels in the diseased tissue by NOX4 inhibitors (NOX4i) or by ROS scavenging with Se or antioxidants results in inhibition of myofibroblast differentiation and, moreover, in dedifferentiation/inactivation of myofibroblasts to a quiescent fibroblast-like phenotype. Similarly, enhancement of NO signaling by administration of the NOS substrate L-arginine, NO-donors (SNP/SNAC), sGC stimulators/activators, or PDE inhibitors (PDEi) maintains the fibroblast phenotype or induces dedifferentiation/inactivation of preexisting myofibroblasts.

Unlike other NOX isoforms, NOX4 is constitutively active with primary regulation occurring at the transcriptional level [59, 65]. NOX4 expression is activated in vascular SMCs and fibroblasts by several cytokines implicated in the pathogenesis of fibrosis, including TGF*β*, angiotensin II, and platelet-derived growth factor [5] and elevated NOX4 levels are observed in tissues bearing hallmarks of fibrosis (Figure 2). For example, *NOX4* mRNA levels specifically correlated with the myofibroblast phenotype in benign prostatic tissue [26]. Similarly, NOX4 expression was higher in pulmonary fibroblasts from patients with IPF compared with controls and correlated with myofibroblast marker expression [66]. In addition, NOX4 was found to be expressed in fibroblastic foci in the lung of IPF patients and two mouse models of pulmonary fibrosis [67]. Recently, high levels of NOX4, which colocalized with *α*-SMA, were observed in liver biopsy samples from patients with autoimmune hepatitis [42]. These observations together with findings from functional studies indicate that elevated NOX4-derived ROS play a critical role in the pathophysiology of numerous fibrotic disorders (Figures 1–3) [26, 40, 67–71]. For example, we demonstrated that NOX4-derived ROS drive myofibroblast differentiation of prostatic fibroblasts in response to TGF*β*1 [26]. Similar findings were observed for cardiac, pulmonary, renal, and adventitial fibroblasts and hepatic stellate cells [39, 40, 42, 66, 67, 72]. In vascular endothelial cells, NOX4 also mediates TGF*β*1-induced cytoskeletal remodeling and maintains the differentiated phenotype of vascular SMCs [73, 74].

Several *in vivo* studies have provided more definitive evidence that NOX4-derived ROS play a direct role in the pathogenesis of fibrosis. For example, inhibition of NOX4 via genetic deletion, antisense oligonucleotides, siRNA, or NOX inhibitors attenuated disease progression in rodent models of pulmonary, renal, and liver fibrosis [42, 67, 82–84].

NOX4 induction appears to contribute to fibrogenesis not via oxidative stress-induced damage [26, 62, 63], but rather by chronic dysregulation of downstream signaling pathways (Figure 2). The precise oxidative target(s) of NOX4-derived ROS that culminate in myofibroblast differentiation in response to TGF*β* remain(s) largely unknown. However, TGF*β*1-induced NOX4-derived ROS have been shown to directly oxidatively inactivate MKP1, a dual specificity MAPK phosphatase that targets JNK and p38 [79]. Consistently, JNK phosphorylation by NOX4-derived ROS was essential for TGF*β*1-induced myofibroblast differentiation of prostatic fibroblasts and cardiomyocyte differentiation of pluripotent embryonal carcinoma cells [26, 80]. Other targets activated by NOX4-derived ROS in fibrogenic signaling cascades include ERK1/2 and Src [39, 81]. Thus, it appears that the NOX4-dependent fibrotic response can be mediated via multiple oxidative targets (Figure 2). Interestingly, activation and release of TGF*β* from its latency association peptide (LAP) are also induced by oxidative modification of LAP with free radicals capable of stimulating TGF*β* expression and secretion in many cell types [29, 85] (Figure 1).

Although NOX4 induction by TGF*β*1 does not typically result in oxidative stress-induced damage in fibroblasts [26, 62, 63], primary alveolar epithelial cells exposed to TGF*β*1 undergo apoptosis in a NOX4-dependent manner [86], an event that can also be indirectly mediated via paracrine release of H_2_O_2_ by activated myofibroblasts [87]. Thus, NOX4 may promote fibrosis not only by driving cytokine-induced fibroblast-to-myofibroblast differentiation but also by impairing epithelial regenerative capacity during wound healing.

In summary, whilst acute induction of NOX4 may be beneficial in inducing the myofibroblast phenotype during wound healing, the persistence of myofibroblasts together with autocrine TGF*β* signaling may result in chronic NOX4 activation and ROS production resulting in a self-perpetuating cycle of myofibroblast differentiation and accumulation, fibrosis, and organ dysfunction (Figure 1). Thus, targeting elevated NOX4-derived ROS either directly via NOX4 inhibition or indirectly by increasing the activity of ROS scavenging enzymes represents a promising therapeutic strategy for the treatment of diverse fibrotic pathologies (Figure 3).

There are numerous ROS-scavenging systems that maintain cellular redox homeostasis; however, of particular interest are the selenium (Se)-dependent enzymes. We observed downregulation of the Se transporter SEPP1 and Se-containing ROS scavengers glutathione peroxidase 3 (GPX3) and thioredoxin reductase 1 (TXNRD1) during TGF*β*1-mediated prostatic myofibroblast differentiation [26]. Moreover, SEPP1 was specifically lost in tumor-associated stroma of prostate cancer patients, indicating reduced activity of ROS scavenging enzymes in the diseases tissue [26]. Se is an essential trace element that is incorporated as selenocysteine into the active sites of GPX3 and TXNRD1 enzymes and required for proper protein folding/function [88]. Consistent with the role of SEPP1 in delivering Se to peripheral tissues for selenoprotein biosynthesis [89, 90], exogenous Se restored expression of GPX3 and TXNRD1 as well as TXNRD1 enzyme activity, depleted TGF*β*1-induced ROS downstream of NOX4 induction, and inhibited myofibroblast differentiation of prostatic fibroblasts [26]. Similarly, exogenous Se also inhibited TGF*β*-mediated myofibroblast transdifferentiation of hepatic stellate cells [91]. Furthermore, we observed that exogenous Se restores morphological and molecular characteristics typical of the fibroblast phenotype to *in vitro *differentiated prostatic myofibroblasts even in the continued presence of the TGF*β* differentiation-inducing stimulus [4]. Similarly, studies employing myofibroblasts from IPF patients and a three-dimensional coculture model of porcine skin fibrosis also demonstrated the potential utility of ROS scavenging in promoting myofibroblast dedifferentiation [92, 93]. Consistently, pharmacological inhibition of NOX4 after induction of liver fibrosis in mice was shown to reduce ROS levels and significantly attenuate fibrosis [42].

Collectively, a large body of *in vitro* and *in vivo *data indicates that myofibroblast differentiation in fibrotic disorders and tumor-reactive stroma is driven by a prooxidant shift in intracellular redox signaling caused by elevated ROS and/or reduced antioxidative potential. NOX4 appears to be the major source of elevated ROS and central mediator of TGF*β*-induced myofibroblast differentiation in diverse tissues. Thus, restoring cellular redox homeostasis by (i) targeting NOX4, (ii) Se supplementation, and/or (iii) application of antioxidants may represent a promising therapeutic strategy for fibrotic disease (Figure 3). Moreover, rather than simply inhibiting myofibroblast differentiation to prevent disease progression, clearing the myofibroblast pool in fibrotic disorders by inducing their dedifferentiation to the nonactivated fibroblast/progenitor phenotype may be a feasible therapeutic strategy that potentially represents a curative treatment.

## 4. Nitric Oxide Signaling in the Regulation of Myofibroblast Differentiation

The free radical NO is an important signaling molecule in a variety of biological processes that is biosynthesized *in vivo* from L-arginine by nitric oxide synthases (NOS), involving the oxidation of NADPH and the reduction of molecular oxygen. NO signaling is mediated via activation of soluble guanylyl cyclase (sGC). The second messenger cyclic guanosine monophosphate (cGMP) that is subsequently generated by sGC regulates the activity of cGMP-dependent protein kinases such as protein kinase G (PKG), cyclic nucleotide phosphodiesterases (PDEs), and cation channels and may have other unknown effects [94].

In terms of fibroblast-to-myofibroblast differentiation, NO signaling appears to be a central pathway associated with the fibroblast phenotype (Figure 3). Treatment of dermal fibroblasts with TGF*β*1 significantly reduced NOS activity and NO levels, whereas the NOS inhibitor *N*_*ω*_-nitro-L-arginine methyl ester (L-NAME) synergistically potentiated TGF*β*1-induced collagen production [95]. Consistently, NOS inhibition or knockout attenuated fibrosis in several animal models [96–99]. We previously demonstrated that the soluble NO donor sodium nitroprusside (SNP) dose-dependently inhibited TGF*β*1-induced myofibroblast differentiation of human prostatic fibroblasts *in vitro* [100]. These findings are in line with suppression of TGF*β*1-induced collagen production by SNP in dermal fibroblasts *in vitro* and attenuation of fibrosis in rodent model systems using the NOS substate L-arginine or the NO donor *S*-Nitroso-*N*-acetylcysteine (SNAC), respectively [95, 98, 101]. Moreover, parallel NO donation and cyclooxygenase inhibition prevented bleomycin-induced lung fibrosis in mice [102].

Since NO activates sGC, increasing sGC activity via NO-independent heme-dependent sGC stimulators represents an alternative approach to enhance NO signaling (Figure 3). Similarly to observations with NO donors, the sGC stimulator BAY 41-2272 inhibited *in vitro* myofibroblast differentiation of cardiac fibroblasts and dermal fibroblasts from healthy subjects and patients with systemic sclerosis [103, 104]. *In vivo *BAY 41-2272 limited disease progression in models of renal, cardiac, and dermal fibrosis [103–106] and similar inhibitory effects were documented for the sGC stimulator riociguat (BAY 63-2521) in rat models [107, 108]. In contrast to sGC stimulators that require the presence of a reduced heme moiety in the prosthetic group of the enzyme, sGC activators can bind to and activate oxidized or heme-deficient sGC [109]. Under conditions of oxidative stress, the heme moiety can be oxidized and lost, rendering sGC no longer responsive to NO. Thus, these heme-independent activators may be beneficial in the treatment of a variety of diseases associated with oxidative stress [109]. Of note, the sGC activator BAY 60-2770 inhibited myofibroblast differentiation in prostatic and dermal fibroblasts (our unpublished observations) and attenuated liver fibrosis in rat models [110], whilst the sGC activator cinaciguat (BAY 58-2667) prevented disease progression in a rat model of chronic renal failure [111].

The fact that treatment with the cell-permeable cGMP analog 8-bromo-cGMP is able to mimic the inhibitory effects of enhanced NO/sGC signaling on myofibroblast differentiation clearly indicates that inhibition is mediated downstream via cGMP [95, 112]. Thus, inhibitors of certain phosphodiesterase isoforms (PDE) represent an additional approach to enhance NO/cGMP signaling. PDEs comprise a superfamily of phosphohydrolases that degrade cellular cGMP and cAMP. PDE type 5 (PDE5), which specifically hydrolyzes cGMP, is the major therapeutic target in erectile dysfunction, and is additionally approved for the treatment of pulmonary arterial hypertension and BPH [113–115]. Increased PDE5 expression was observed in anti-Thy1-induced mesangial proliferative glomerulonephritis in rats and PDE5 inhibition showed beneficial antiproliferative and antifibrotic effects* in vivo*, indicating an active role of PDE5 in fibrogenesis [116]. We previously demonstrated that pharmacological inhibition or shRNA-mediated silencing of PDE5 significantly attenuated TGF*β*1-induced myofibroblast differentiation of prostatic fibroblasts *in vitro* [100]. Likewise, PDE5 inhibition prevented myofibroblast differentiation in fibroblasts form Peyronie's disease plaques *in vitro* and counteracted fibrosis in TGF*β*1-induced Peyronie's disease-like plaques in rats [117, 118]. Moreover, in lung fibroblasts PDE5 inhibition in combination with the sGC activator cinaciguat attenuated myofibroblast differentiation [41].

Similarly to exogenous Se, we recently reported that PDE5 inhibition in *in vitro* differentiated prostatic myofibroblasts restored morphological and molecular characteristics typical of the fibroblast phenotype, indicating that enhancement of NO signaling not only prevents but also might reverse fibrosis [119]. Consistently, the NO donor SNAC induced dedifferentiation of activated hepatic stellate cells *in vitro* [120] and *in vivo* sGC stimulation by BAY 41-8543 decreased tubulointerstitial fibrosis after relief of unilateral ureteral obstruction in rats [121]. Similarly, BAY 41-2272 reduced established fibrosis in modified mouse models of dermal fibrosis [34] and PDE5 inhibition reduced myofibroblast numbers and total size of preformed TGF*β*1-induced Peyronie's disease-like plaques in rats [117]. Of note, various PDE5 inhibitors selectively increased the apoptotic index in TGF*β*1-induced Peyronie's disease-like plaques in rats [117, 118], indicating clearance of myofibroblasts by apoptosis upon enhancement of NO signaling.

Collectively, findings from *in vitro* and *in vivo* model systems indicate that the fibroblast phenotype is maintained by NO signaling and that myofibroblast differentiation is associated with an attenuation/inhibition of the NO/sGC/cGMP signaling cascade, while stimulation of NO signaling is capable of even reverting myofibroblast differentiation. Thus, enhancement of NO signaling by NO donors, stimulators, and activators of sGC or inhibition of cGMP degradation via PDE inhibitors might be of therapeutic benefit for patients suffering from degenerative fibrotic disease (Figure 3).

## 5. Crosstalk between ****NOX4/H**_**2**_**O**_**2**_** and NO Signaling Networks in the Regulation of Myofibroblast Differentiation

The fact that elevated NO signaling attenuates and reverses myofibroblast differentiation while NOX4-derived ROS play a key role in driving differentiation in response to TGF*β* indicates that the fibroblast/myofibroblast phenotype is regulated via crosstalk between both signaling pathways. The main ROS effector of NOX4 is H_2_O_2_ [59–61]; however, by virtue of its catalytic structure [122] its primary product like other NOX isoforms is superoxide (see chapter 3) [59–61]. Even assuming that NOX4-derived superoxide undergoes rapid dismutation, residual superoxide could potentially cross-react with NO signaling; for example, superoxide can react with NO generating peroxynitrite (ONOO^−^), thereby depleting NO levels [123]. In addition, superoxide can oxidize the critical nitric oxide synthase (NOS) cofactor tetrahydrobiopterin (BH_4_) leading to NOS uncoupling and superoxide generation rather than NO production [124]. Indeed, in some models, NOX4 has been implicated in the generation of peroxynitrite and subsequent NOS uncoupling [125–128]. However, since NOX4 is primarily associated with constitutive H_2_O_2_ production [60, 61], which unlike superoxide does not appear to react directly with NO, any opposing regulation of TGF*β*-induced myofibroblast differentiation by NO and NOX4-derived ROS signaling presumably predominantly occurs via distinct mechanisms (summarized Figure 3).

There are several mechanisms by which NOX4-derived H_2_O_2_ may affect NO signaling. H_2_O_2_ impaired NO production in porcine aortic endothelial cells possibly via direct oxidative inactivation of eNOS cofactors [129]. Moreover, H_2_O_2_ decreased sGC expression and consequently NO-dependent cGMP generation in pulmonary arterial SMCs from lambs with persistent pulmonary hypertension of the newborn and in rat aortic SMCs or freshly isolated vessels [130, 131]. H_2_O_2_ or PTP inhibitors promoted tyrosine phosphorylation of the beta 1 subunit of sGC, presumably via Src-like kinases. Since c-Src-dependent phosphorylation of sGC has been shown to attenuate sGC activity and cGMP formation [132, 133], these data suggest that elevated NOX4-derived H_2_O_2_ during myofibroblast differentiation may inactivate PTPs and/or activate Src kinase, leading to sGC phosphorylation and consequently reduced cGMP formation.

Additionally, NOX4-derived H_2_O_2_ and NO signaling may interact via common cofactors. Both NOS and NOX require NADPH as an electron donor for enzyme activity. Since NOX4 induction is an early event during TGF*β*1-mediated differentiation [26, 40], NADPH consumption/depletion due to elevated NOX4 activity may attenuate NOS activity and consequently NO signaling. Furthermore, opposing interaction may occur via mutually exclusive modification of NOX/NO target proteins. For example, NO activates sarco/endoplasmic reticulum Ca2+ ATPase (SERCA) via *S*-glutathiolation on cysteine 674, while induction of NOX4 via TGF*β*1, exposure to H_2_O_2_, or high glucose resulted in SERCA oxidation of the same thiol group that inhibited NO-mediated *S*-glutathiolation [134–136].

Taken together these findings clearly indicates that H_2_O_2_ and NO appear to interact in a functionally opposing manner during myofibroblast differentiation via multiple mechanisms, whereby TGF*β*1-mediated induction of NOX4-derived H_2_O_2_ leads to downregulation of NO signaling and thereby promotes fibroblast-to-myofibroblast differentiation. Consistently, TGF*β*1 significantly decreased NO production in dermal fibroblasts [95]. Thus, stimulating sGC generation and/or inhibiting cGMP degradation potentially counteract ROS-mediated inactivation of NO signaling to consequently prevent and reverse myofibroblast differentiation. Of note, enhancing cGMP levels inhibited and reversed differentiation without impairing NOX4 mRNA induction by TGF*β*1 (our unpublished observations) [119], indicating that NO signaling acts downstream of NOX4-derived H_2_O_2_ production. Since treatment with 8-bromo-cGMP is sufficient to inhibit myofibroblast differentiation [95], the H_2_O_2_-counteracting effects of elevated NO signaling appear to be mediated via downstream cGMP-dependent mechanisms and not via the NO radical *per se*.

## 6. Clinical Implications

In order to develop broadly effective antifibrotic therapies, it will be necessary to identify common features of different fibrotic disorders that affect distinct tissues and/or are initiated by different stimuli (e.g., chronic scarring of the liver due to hepatitis versus the tumor-associated reactive stromal response to prostate cancer). However, in some cases it may be necessary/advantageous to identify tissue-specific signaling mechanisms/inducers whose specific targeting is less likely to be associated with adverse side effects on healthy tissues. The observation that fibroblasts and myofibroblasts are interconvertible phenotypes, with phenotypic switching apparently regulated via crosstalk between NOX4/H_2_O_2_ and NO signaling, has significant clinical implications. Targeting redox signaling, for example, via inhibitors of NOX4 or PDE5, antioxidants such as Se or enhancers of NO signaling, represents a promising therapeutic strategy to modulate the fibroblast/myofibroblast ratio in pathological conditions such as degenerative fibrotic diseases (Figure 3).

TGF*β* unequivocally plays a central role in fibrogenesis in diverse tissues and organs. However, given its essential role in a wide range of fundamental cellular functions, there are concerns that systemic approaches directly targeting TGF*β* for the treatment of fibrotic conditions will potentially exert undesirable toxic effects [51, 137]. Indeed, this was the case in the CAT-192 clinical trial [138]. Nonetheless, several clinical trials on fibrosis employing anti-TGF*β* agents have been completed and several others are underway. Unfortunately, to date, these trials have largely yielded disappointing results despite promising *in vitro* observations (reviewed recently [139, 140]).

Induction of NOX4-derived H_2_O_2_ and reduced NO signaling are apparently central downstream components of TGF*β*-mediated myofibroblast differentiation in diverse tissues and organs. Thus, therapeutic targeting of redox homeostasis in degenerative fibrotic diseases might also be expected to elicit broad and undesirable effects. However, it should be noted that, unlike TGF*β* that drives myofibroblast differentiation and fibrosis via Smad-dependent and -independent pathways [51, 141], NOX4 does not modulate noncanonical signaling by TGF*β*1 in prostatic fibroblasts [26, 40, 65]. Moreover, Nox4 knockout animals display no obvious basal phenotype and a dual NOX1/NOX4 inhibitor was well tolerated in animal models and in phase I clinical trials [42, 57]. Furthermore, PDE(5) inhibitors are clinically employed for a variety of conditions and have a history of safe use with minimal side effects in humans [113–115].

Despite intense research efforts, there currently remains no NOX4-specific inhibitor and several attempts to generate peptides that disrupt NOX4 function have been unsuccessful with the authors concluding that, unlike other NOX isoforms, NOX4 exists in a tightly assembled and active conformation, which cannot be disrupted by conventional means [142, 143]. Nonetheless, several studies have successfully employed a dual NOX1/NOX4 inhibitor GKT137831, which showed promising results in mouse models of liver fibrosis and hypoxia-induced pulmonary hypertension [42, 57, 84, 144] and is currently entering a phase II clinical trial for diabetic nephropathy. Recently, attenuation of NOX4-dependent ROS signaling and fibrosis by sodium hydrosulfide and nifedipine (an L-type dihydropyridine calcium channel blocker) was reported in rodent models of cardiac fibrosis [145, 146]. However, it remains to be determined whether these compounds inhibit NOX4 in a specific and isoform-selective manner or exert their effects via nonspecific and nonselective mechanisms.

Given that the signaling potential of NOX4-derived ROS is regulated by antioxidant systems, enhancing the activity of ROS scavenging enzymes may represent an alternative potential therapeutic strategy (Figure 3). Animal and human clinical data clearly demonstrate that Se deficiency or supplementation increases or reduces tumor incidence, respectively [147–152]. In addition, however, serum Se levels have been reported to be lower in patients with several different fibrotic disorders, including systemic sclerosis, primary Raynaud's phenomenon, and oral submucous fibrosis [153, 154]. Unfortunately, there are few studies investigating the potential therapeutic benefit of Se supplementation in degenerative fibrotic disease. Although proof-of-principle is provided by reports that exogenous Se was shown to decrease hepatic fibrosis in mice [155], Se deficiency promoted thyroid fibrosis in a TGF*β*-dependent manner in rats [156]. However, there may be a potential increased risk of diabetes with Se supplementation [157]; thus further studies are required to better understand the biological effects of Se to allow its use in the prevention and treatment of degenerative fibrotic disease.

Inhibitors of PDE5 are clinically approved for the treatment of erectile dysfunction, pulmonary arterial hypertension, and BPH [113–115] and apparently have significant efficacy in Raynaud's phenomenon secondary to systemic sclerosis [158, 159]. The sGC stimulator riociguat significantly improved primary and secondary endpoints in recently presented phase III clinical trials in patients with pulmonary arterial hypertension and chronic thromboembolic pulmonary hypertension [160, 161]. Although the clinical development of the heme-independent sGC activators cinaciguat and ataciguat stopped in clinical phase II trials, the perspective to specifically activate oxidated, heme-free sGC generated by the influence of oxidative stress, seems very promising of offering novel therapies for various disorders associated with oxidative stress and several second-generation sGC activators have been developed recently [162, 163].

Due to the presence of multiple NOX, PDE, and GC isoforms, modulation of NOX4, PDE5, and/or sGC activities may permit continued physiological H_2_O_2_ and NO signaling. In addition, the fact that these enzymes belong to multimembered families may be clinically exploited to selectively target tissue or disease-specific isoforms. For example, selective targeting of PDE1A, that appears to play a critical role in cardiac fibrosis, led to regression of cardiac remodeling in rodents [164].

## 7. Conclusions

Fibrogenesis is widely considered the result of a dysregulated wound healing response. In particular, failure of the wave of myofibroblast apoptosis during wound healing combined with an autocrine feed-forward loop of TGF*β* production leads to the development and persistence of large numbers of myofibroblasts, a hallmark of fibrotic disorders (Figure 1). TGF*β* plays a key role in initiating myofibroblast differentiation from diverse precursors, most importantly fibroblasts, in a variety of organs and tissues. A large body of *in vitro* and *in vivo* data indicates that TGF*β*-induced myofibroblast differentiation is driven via induction of NOX4-derived ROS (Figure 2) and supported by the concomitant downregulation of Se-dependent ROS scavenging enzymes. The resulting prooxidant shift in redox homeostasis modulates redox-sensitive signaling cascades leading to myofibroblast differentiation (Figure 2). Interestingly, myofibroblast differentiation appears to be subject to opposing regulation via complex interplay between NOX4-derived H_2_O_2_ and NO signaling. Whilst TGF*β* and NOX4-derived H_2_O_2_ attenuate NO signaling by impairing NOS and sGC activities and thus relieve inhibition of myofibroblast differentiation by NO, enhancement of NO signaling prevents TGF*β*-induced myofibroblast differentiation (Figure 3). Moreover, targeting NOX4 or enhancing NO signaling induces the dedifferentiation/reversal of preexisting myofibroblasts to a quiescent fibroblast phenotype and ameliorates fibrosis *in vivo* indicating that fibroblasts and myofibroblasts are interconvertible phenotypes. Thus, pharmacological interference of these redox signaling processes to restore the physiological fibroblast:myofibroblast ratio offers a promising strategy for the treatment of fibrosis and degenerative fibrotic diseases. Therapeutic intervention could be potentially achieved at multiple levels, for example, by (i) targeting NOX4 directly using specific inhibitors, (ii) indirectly inhibiting NOX4 using antioxidants or Se to scavenge ROS/H_2_O_2_, (iii) enhancing NO signaling via NO-donors, stimulators/activators of sGC, and/or (iv) preventing cGMP degradation using PDE inhibitors. It is hoped that the recent findings summarized herein can be applied and translated into effective therapeutic strategies for the treatment of debilitating fibrotic disorders.

## Abbreviations

*α*-SMA: Alpha smooth muscle cell actin
BPH: Benign prostatic hyperplasia
cAMP: Cyclic adenosine monophosphate
cGMP: Cyclic guanosine monophosphate
ECM: Extracellular matrix
IPF: Idiopathic pulmonary fibrosis
LAP: Latency associated peptide
L-NAME: *N*_*ω*_-Nitro-L-arginine methyl ester
MAPK: Mitogen-associated protein kinase
NO: Nitric oxide
NOS: Nitric oxide synthase
NOX: NADPH oxidase
PDE: Phosphodiesterase
PKG: Protein kinase G
PTP: Protein tyrosine phosphatase
ROS: Reactive oxygen species
Se: Selenium
sGC: Soluble guanylyl cyclase
SMC: Smooth muscle cell
SNAC: *S*-Nitroso-*N*-acetylcysteine
SNP: Sodium nitroprusside
TGF*β*: Transforming growth factor beta.
